# A rare case of duodenojejunal intussusception causing biliary obstruction during pregnancy

**DOI:** 10.1093/jscr/rjaf686

**Published:** 2025-09-08

**Authors:** Salma El Aouadi, Kaoutar Maslouhi, Soukaina Bahha, Zakaria Guetmy, Amina B El Alaoui, Zaynab I Houssaini, Omar El Aoufir, Laila Jroundi, Ittimade Nassar, Ola Messaoud

**Affiliations:** Department of Radiology, Ibn Sina University Hospital Center, Avenue Ibn Rochd, Agdal District, Rabat 10000, Rabat-Salé-Kénitra Region, Morocco; Department of Radiology, Ibn Sina University Hospital Center, Avenue Ibn Rochd, Agdal District, Rabat 10000, Rabat-Salé-Kénitra Region, Morocco; Department of Radiology, Ibn Sina University Hospital Center, Avenue Ibn Rochd, Agdal District, Rabat 10000, Rabat-Salé-Kénitra Region, Morocco; Emergency Surgical Department, Ibn Sina University Hospital Center, Avenue Ibn Rochd, Agdal District, Rabat 10000, Rabat-Salé-Kénitra Region, Morocco; Emergency Surgical Department, Ibn Sina University Hospital Center, Avenue Ibn Rochd, Agdal District, Rabat 10000, Rabat-Salé-Kénitra Region, Morocco; Department of Radiology, Ibn Sina University Hospital Center, Avenue Ibn Rochd, Agdal District, Rabat 10000, Rabat-Salé-Kénitra Region, Morocco; Department of Radiology, Ibn Sina University Hospital Center, Avenue Ibn Rochd, Agdal District, Rabat 10000, Rabat-Salé-Kénitra Region, Morocco; Department of Radiology, Ibn Sina University Hospital Center, Avenue Ibn Rochd, Agdal District, Rabat 10000, Rabat-Salé-Kénitra Region, Morocco; Department of Radiology, Ibn Sina University Hospital Center, Avenue Ibn Rochd, Agdal District, Rabat 10000, Rabat-Salé-Kénitra Region, Morocco; Department of Radiology, Ibn Sina University Hospital Center, Avenue Ibn Rochd, Agdal District, Rabat 10000, Rabat-Salé-Kénitra Region, Morocco

**Keywords:** duodenojejunal intussusception, pregnancy, biliary obstruction, acute abdominal pain

## Abstract

Intussusception is an uncommon cause of intestinal obstruction in adults and rarely encountered during pregnancy. Duodenal intussusception is particularly rare due to the fixed position of the duodenum. We report a unique case of duodenojejunal intussusception in a pregnant woman at 28 weeks of gestation, who presented with symptoms mimicking acute pancreatitis complicated by biliary tract obstruction. To our knowledge, this is the first description of such a presentation without an identifiable lead point, illustrating the diagnostic value of imaging and the importance of considering rare etiologies in the differential diagnosis of abdominal pain during pregnancy.

## Introduction

Intussusception is the invagination of one bowel segment into another, typically with proximal segment telescoping into the distal [1]. Duodenal involvement is rare because of its fixed retroperitoneal location and is usually linked to a structural lead point [1]. In pregnancy, evaluating abdominal pain is challenging because of overlapping symptoms and limitations of imaging modalities to avoid fetal radiation exposure [2]. We report a rare case of duodenojejunal intussusception during the third trimester, complicated by biliary obstruction and occurring without an identifiable lead point.

## Case report

A 32-year-old woman, with no significant medical or surgical history, presented at 28 weeks of gestation with a 15-day history of persistent vomiting, associated with progressively worsening epigastric pain over the preceding 4 days. The pain was transfixing in nature, radiating to the back, and unresponsive to analgesics. She also reported jaundice for 2 days. Abdominal examination revealed epigastric tenderness without signs of peritoneal irritation.

Laboratory tests showed evidence of cholestasis and cytolysis, with elevated total bilirubin and serum lipase, raising suspicion of acute pancreatitis.

**Figure 1:**
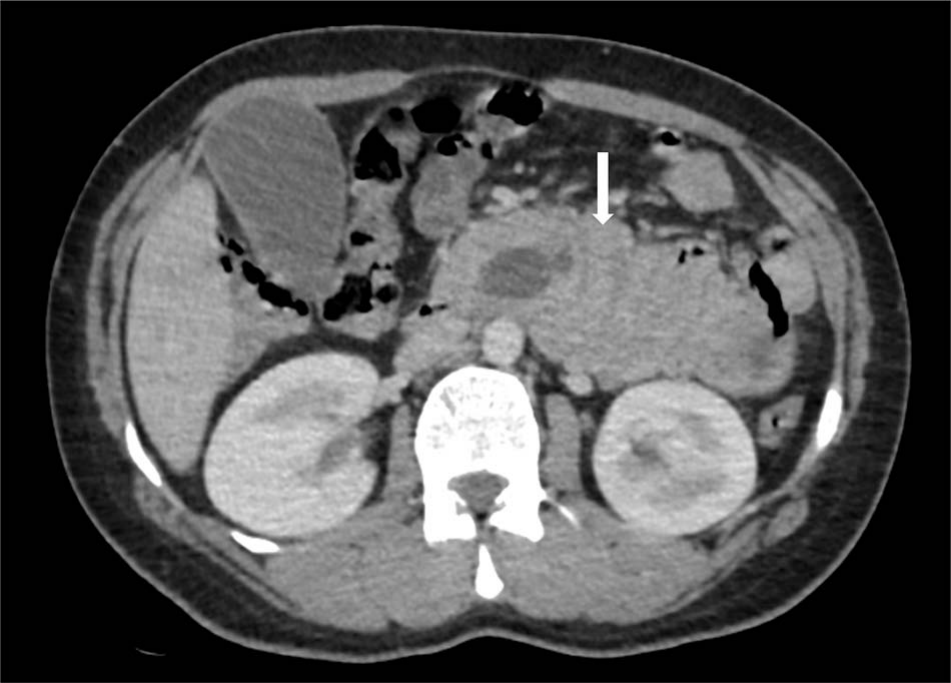
Axial contrast-enhanced CT image showing a duodenojejunal intussusception (arrow), originating from the fourth portion of the duodenum and involving invagination of a proximal jejunal loop.

**Figure 2:**
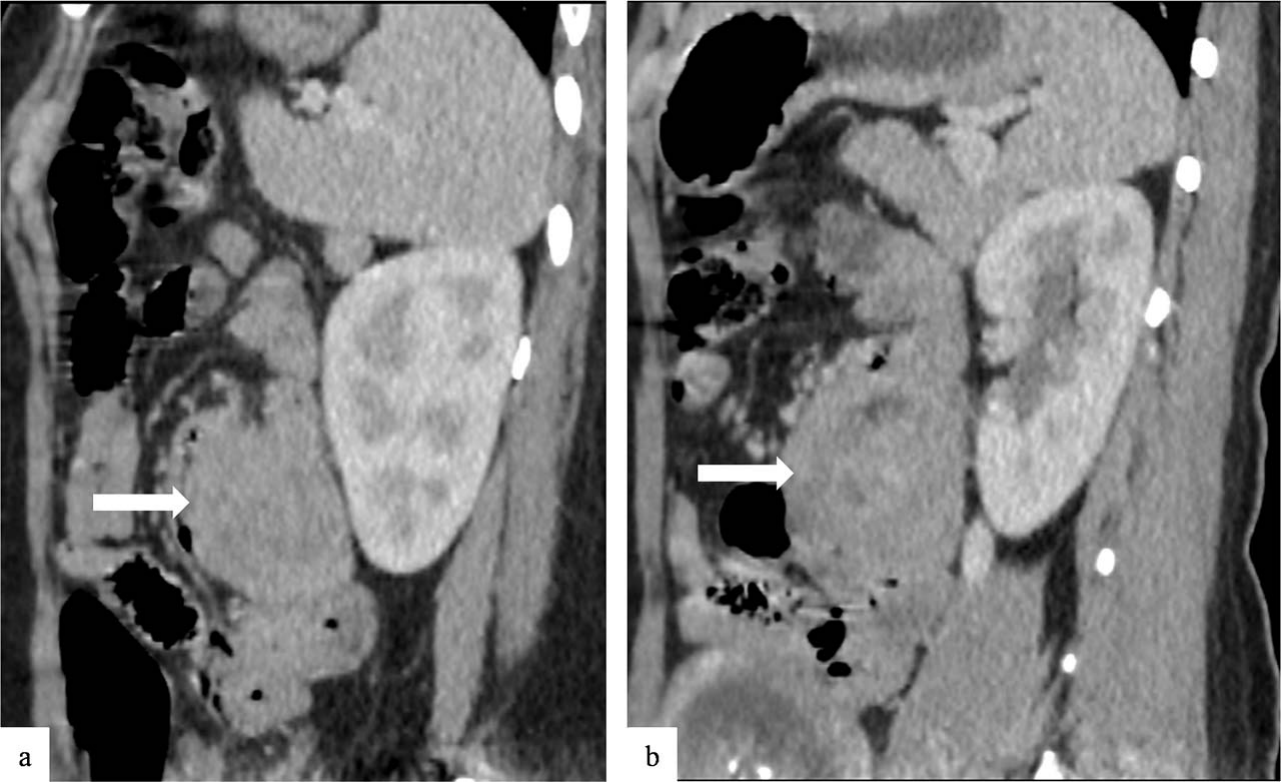
Sagittal contrast-enhanced CT images (a, b) showing the duodenojejunal intussusception with a pseudokidney appearance (arrows).

**Figure 3:**
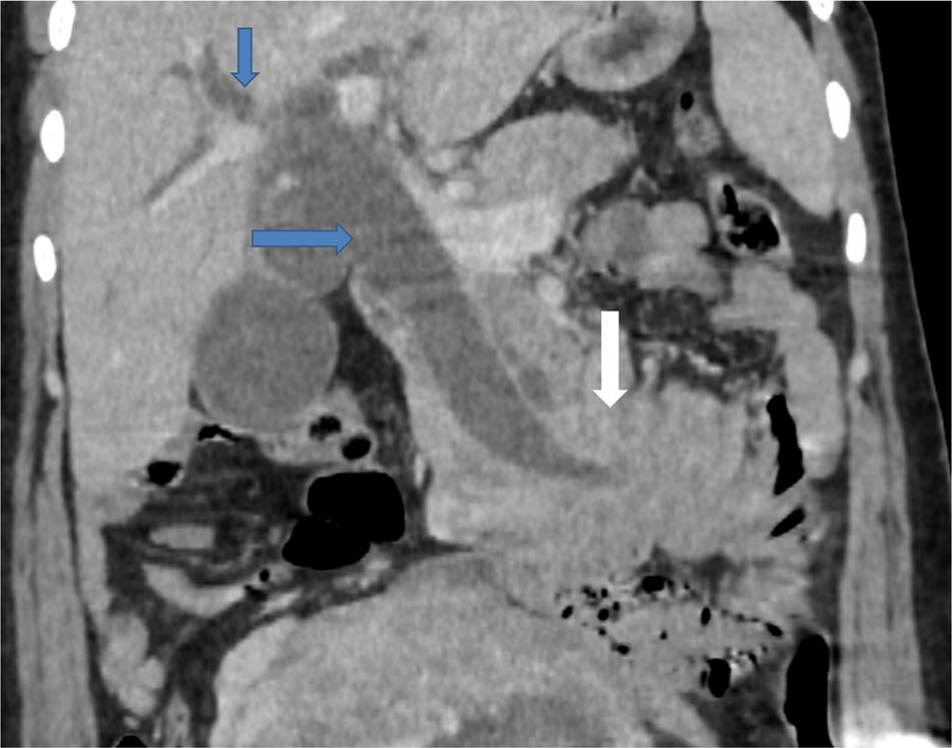
Coronal contrast-enhanced CT image showing the intussusception causing traction on the distal common bile duct (arrow at the bottom), resulting in significant upstream dilatation of the extrahepatic and intrahepatic bile ducts (two arrows at the top).

Due to the acute presentation and unavailability of emergency magnetic resonance imaging (MRI), a contrast-enhanced computed tomography (CT) scan of the abdomen was performed with strict fetal protection measures (lead apron, low-dose protocol). The pancreas appeared normal with no signs of inflammation, necrosis, or peripancreatic fluid. Unexpectedly, the scan revealed a duodenojejunal intussusception, likely originating from the fourth portion of the duodenum (D4), involving invagination of a proximal jejunal loop (Fig. 1). A typical pseudokidney sign was observed on sagittal reconstructions (Fig. 2). There was no identifiable lead point such as a mass or polyp. The intussusception was seen tractioning the distal common bile duct, leading to marked extra- and intrahepatic bile duct dilatation (Fig. 3).

**Figure 4:**
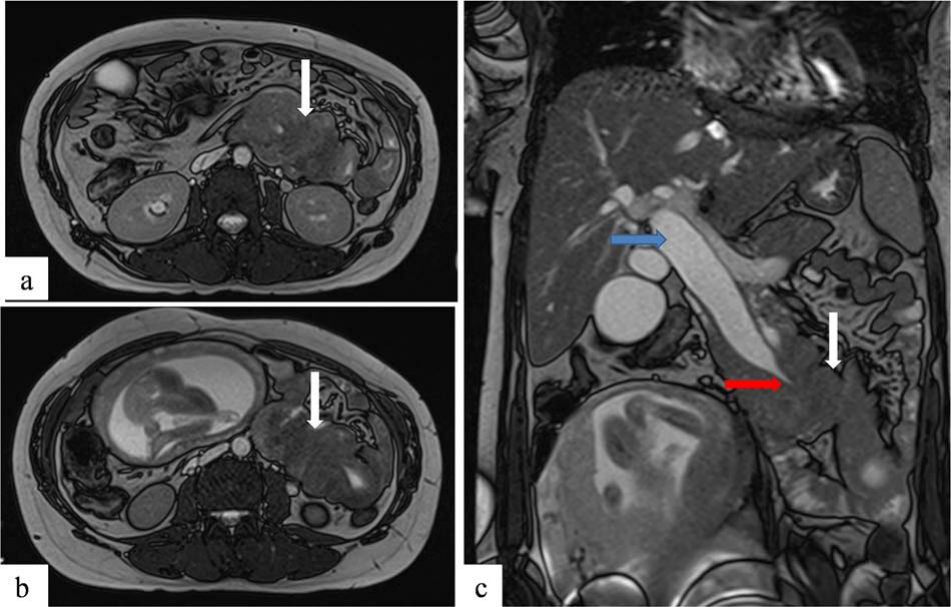
Axial (a, b) and coronal (c) T2-weighted TrueFISP MR images confirming the duodenojejunal intussusception (white arrows) and showing inclusion of the terminal bile duct within the intussuscepted segment (red arrow), resulting in significant upstream biliary dilatation (blue arrow).

**Figure 5:**
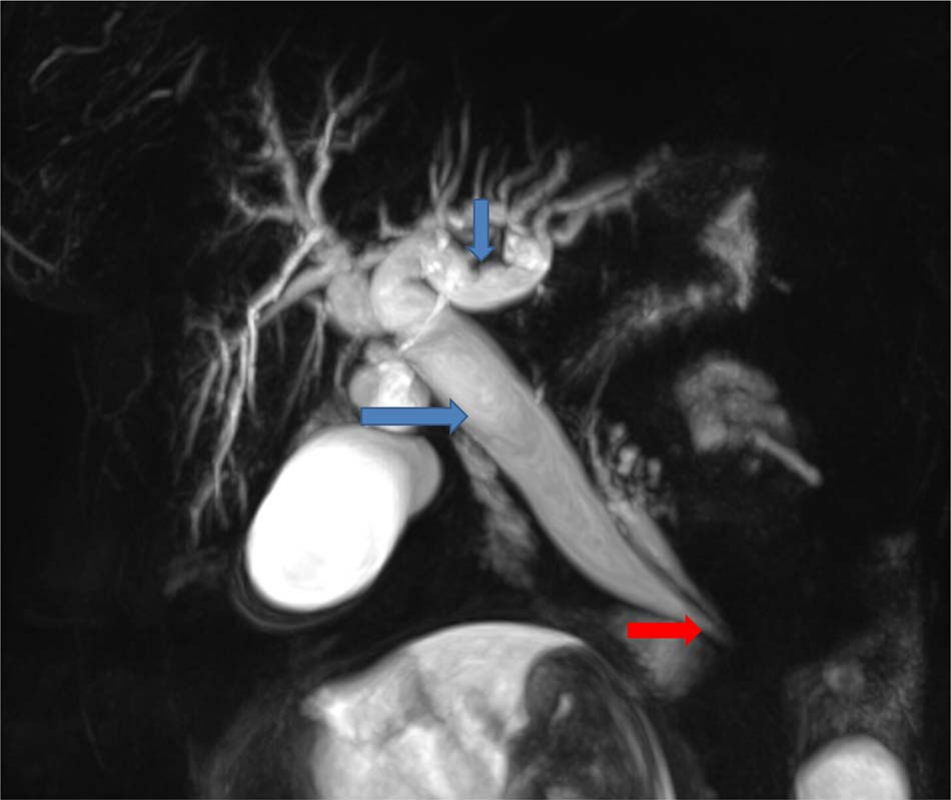
Coronal MR cholangiopancreatographic image showing an ectopic biliopancreatic confluence draining into the fourth portion of the duodenum (arrow at the bottom), which likely predisposed the biliary system to traction during the intussusception, resulting in upstream biliary duct dilatation (two arrows at the top).

To better assess the biliary anatomy and clarify the cause of obstruction, a MR cholangiopancreatography (MRCP) was performed 2 days later. It confirmed the duodenojejunal invagination and demonstrated the involvement of the terminal bile duct within the intussuscepted segment (Fig. 4). An ectopic biliopancreatic confluence was also identified, draining into the fourth portion of the duodenum, likely predisposing the bile duct to traction and resulting in upstream dilation (Fig. 5).

The patient was hospitalized for close clinical and obstetrical monitoring. Supportive treatment included intravenous fluids and antiemetic therapy. Liver function tests gradually improved, and symptoms resolved under conservative management. Given the absence of alarming signs such as bowel ischemia or systemic infection, and the gestational age of 28 weeks, surgical management was deferred. She remained clinically stable and was discharged after 7 days, with outpatient follow-up. Obstetric monitoring continued uneventfully, with normal fetal growth and activity.

## Discussion

Intussusception is defined as the invagination of a proximal bowel segment and its mesentery into an adjacent distal segment [1]. It is common in children but rare in adults, representing 0.03% of hospital admissions, and even more uncommon during pregnancy [2].

**Figure 6:**
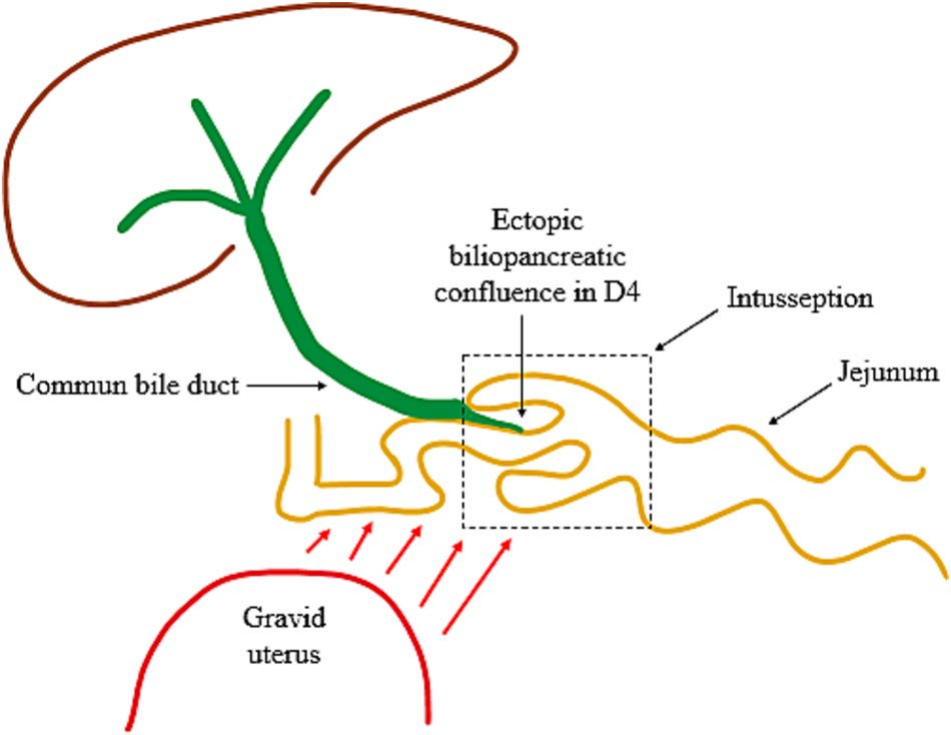
Illustration of the intussusception mechanism and biliary traction due to mechanical displacement from the gravid uterus and an ectopic biliopancreatic confluence in the fourth portion of the duodenum (D4).

Intussusception is classified based on the involved segments: enteroenteric, colocolonic, or enterocolonic [3]. The mechanism is unclear but likely involves abnormal peristalsis [3]. Enteric intussusception is the most frequent form in adults, while duodenal involvement is rare due to the duodenum’s fixed position [3]. It is usually linked to a lead point such as lipomas, hamartomatous polyps, or proliferative lesions like Brunner’s gland hamartoma or hyperplasia [4]. This case illustrates a rare duodenojejunal intussusception in a pregnant woman. Without a clear lead point, pregnancy-related physiological changes—like mechanical displacement of the bowel by the gravid uterus—may disrupt normal peristalsis and promote intussusception. A schematic illustration of this proposed mechanism is shown in Fig. 6.

The clinical symptoms of duodenal intussusception are often non-specific and can be mistaken, especially during pregnancy, when nausea, vomiting, pain, and constipation are common. Moreover, the gravid uterus displaces the bowel, complicating the examination and delaying diagnosis [2]. Typical signs include epigastric pain, an abdominal mass, gastrointestinal bleeding, or signs of enteral obstruction. The biliary and pancreatic systems are rarely involved [4, 5]. Our case was unusual due to biliary obstruction caused by intussusception, with few similar cases reported [6–10].

Multiple imaging modalities are used to diagnose intussusception. In pregnancy, fetal safety guides the choice of technique [10]. Ultrasound and MRI are preferred due to the risks of radiation exposure from CT, which is commonly used otherwise [10]. Ultrasound is accessible and effective, showing characteristic signs like the target and pseudo-kidney signs, but can be limited by obesity, bowel gas, and operator skill [2]. CT remains the most reliable method, revealing the bowel-within-the-bowel appearance, manifested by the target sign or a sausage-shaped soft tissue mass [7]. The lead point, if present, can often be identified [2]. It also assesses disease extent and complications such as ischemia or perforation [2]. Another imaging modality that can be considered is MRI, especially in pregnant women and in cases with biliary tract dilatation [2]. On fluid-sensitive sequences, intussusception appears as hyperintense intraluminal fluid surrounded by the low-to-intermediate signal intensity of the bowel wall, often associated with perienteric edema [2, 7].

Although the management of intussusception in adults remains a subject of debate, surgical resection is generally preferred for small-bowel intussusception due to the common presence of a pathological lead point and the challenges associated with non-surgical reduction [6]. In cases of idiopathic forms, some authors recommend performing a simple reduction, provided that bowel ischemia and perforation have been ruled out [4].

To our knowledge, this is the first reported case of duodenojejunal intussusception causing biliary obstruction during pregnancy without a structural lead point. It highlights the importance of thorough evaluation of unusual abdominal or biliary symptoms in pregnant women, as these may signal rare but serious conditions requiring prompt diagnosis and management.

## Conclusion

Duodenojejunal intussusception during pregnancy is extremely rare and can present with nonspecific symptoms resembling common gestational or gastrointestinal disorders. This case underscores the importance of considering uncommon causes of abdominal pain in pregnant patients and highlights the key role of imaging, especially MRI, for timely and accurate diagnosis while minimizing fetal risk. Early detection is crucial for optimal management and avoiding unnecessary interventions.
